# 3D model of the geometric nest structure, the “mystery circle,” constructed by pufferfish

**DOI:** 10.1038/s41597-022-01466-4

**Published:** 2022-07-04

**Authors:** Hiroshi Kawase, Yuki Kitajima, Daisuke Iwai

**Affiliations:** 1 Coastal Branch of Natural History Museum and Institute, Chiba, 123 Yoshio, Katsuura, Chiba 299-5242 Japan; 2 Graduate School of Engineering Science, Osaka University, 1-3 Machikaneyama, Toyonaka, Osaka, 560-8531 Japan; 3 Present Address: Saiin Sanzocho, Ukyo, Kyoto 615-0021 Japan

**Keywords:** Behavioural ecology, Animal behaviour, Ichthyology, Fluid dynamics, Marine biology

## Abstract

A small pufferfish, *Torquigener albomaculosus*, is known to construct an elaborate geometric circular structure, which has been referred to as a “mystery circle,” with a diameter of ~2 m in the sand of the seabed. We reconstructed a 3D model of this structure for the first time using a “structure from motion” (SfM) algorithm. The mystery circle constructed by the pufferfish may have potential applications for biomimetics similar to the structures constructed by termites and prairie dogs. To support the significance of its structural characteristics, it was observed that the water passing through the valley upstream always gathers in the center of the structure, regardless of the direction of water flow. Furthermore, it has the function of extracting fine-grained sand particles from the valleys and directing these to the center. Computational fluid analysis can be performed immediately using the quantified 3D data, and the structural features of the mystery circle is expected to be applied in a wide range of fields, such as architecture and engineering, via biomimetics.

## Abstract (summary)

**Table:** 

Measurement(s)	3D structure
Technology Type(s)	SfM
Factor Type(s)	3D model
Sample Characteristic - Organism	Tetraodontidae • *Torquigener albomaculosus* • pisces
Sample Characteristic - Environment	sandy seabed • underwater • subtropical sea
Sample Characteristic - Location	Amami-Oshima Island • Japan • Pacific Ocean

## Background & Summary

Wild animals construct various types of structures that are adaptive to their life and reproduction. For example, termites that inhabit the African savanna use soil to construct a huge mound that reaches 10 m in height; they produce hollows and holes in these mounds to allow air ventilation, thereby keeping the internal temperature constant^1^. In addition, prairie dogs inhabiting the North American prairie dig vertically and horizontally extending burrows in the ground that they use for shelter and rearing offspring; these burrows have multiple entrances, some of which are chimney-shaped to improve ventilation efficiency^2^. In the field of biomimetics, researchers apply the principles of animal-created structures in applications useful to humans^3^.

**Fig. 1:**
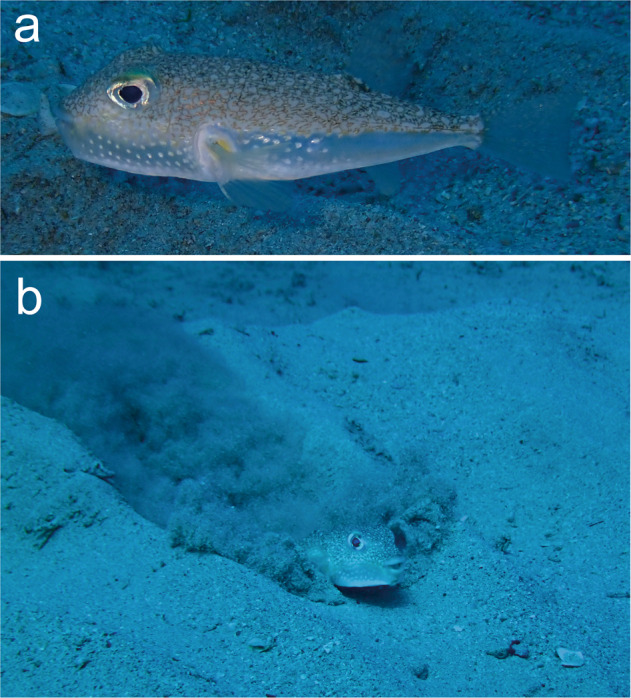
The white-spotted pufferfish *Torquigener albomaculosus*. Lateral view of a male (**a**), and male digging behaviour on the seabed while rolling up fine sand particles (**b**).

The white-spotted pufferfish *Torquigener albomaculosus* (Pisces: Tetraodontidae) is a relatively small species that grows to ~10 cm in total total length (Fig. 1). Male *T. albomaculosus* individuals construct an intricate geometric circular structure, known as the “mystery circle,” with a diameter of 2 m in the sand of the seabed;^4^ the discovery of these structures has fascinated researchers and the general public worldwide. The male pufferfish digs the sand on the seabed with its fins and body while swimming straight ahead toward the centre from different directions, and a circular structure composed of radially aligned peaks and valleys was constructed. Finally, the male creates a maze-like pattern by flapping its anal fin on the bottom of the central zone^4^. Thus, the male completes the circular structure by himself. Furthermore, we discovered that the earliest stage of the mystery circle is composed of dozens of irregular depressions, which might function as landmarks for the formation of the radial patterns^5^. By accumulating observations of pufferfish behaviour, we were able to conduct a computer simulation including the swimming trajectory of the pufferfish extracted from video images wherein they constructed the circular structure. This simulation revealed that an elaborate circular geometric pattern is inevitably formed if the pufferfish repeats the digging behavior on the seabed using simple rules^6^. We also observed the reproductive behaviour of the pufferfish and found that they consistently breed in a semilunar cycle from spring to summer. Each male constructs a mystery circle and spawns with multiple females on the nest, and the male cares for the eggs alone until they hatch. Some of the elements of the circular structure, i.e., its size, symmetry, ornaments, and maze-like pattern, might be important factors in terms of female mate choice^4,7^.

**Fig. 2:**
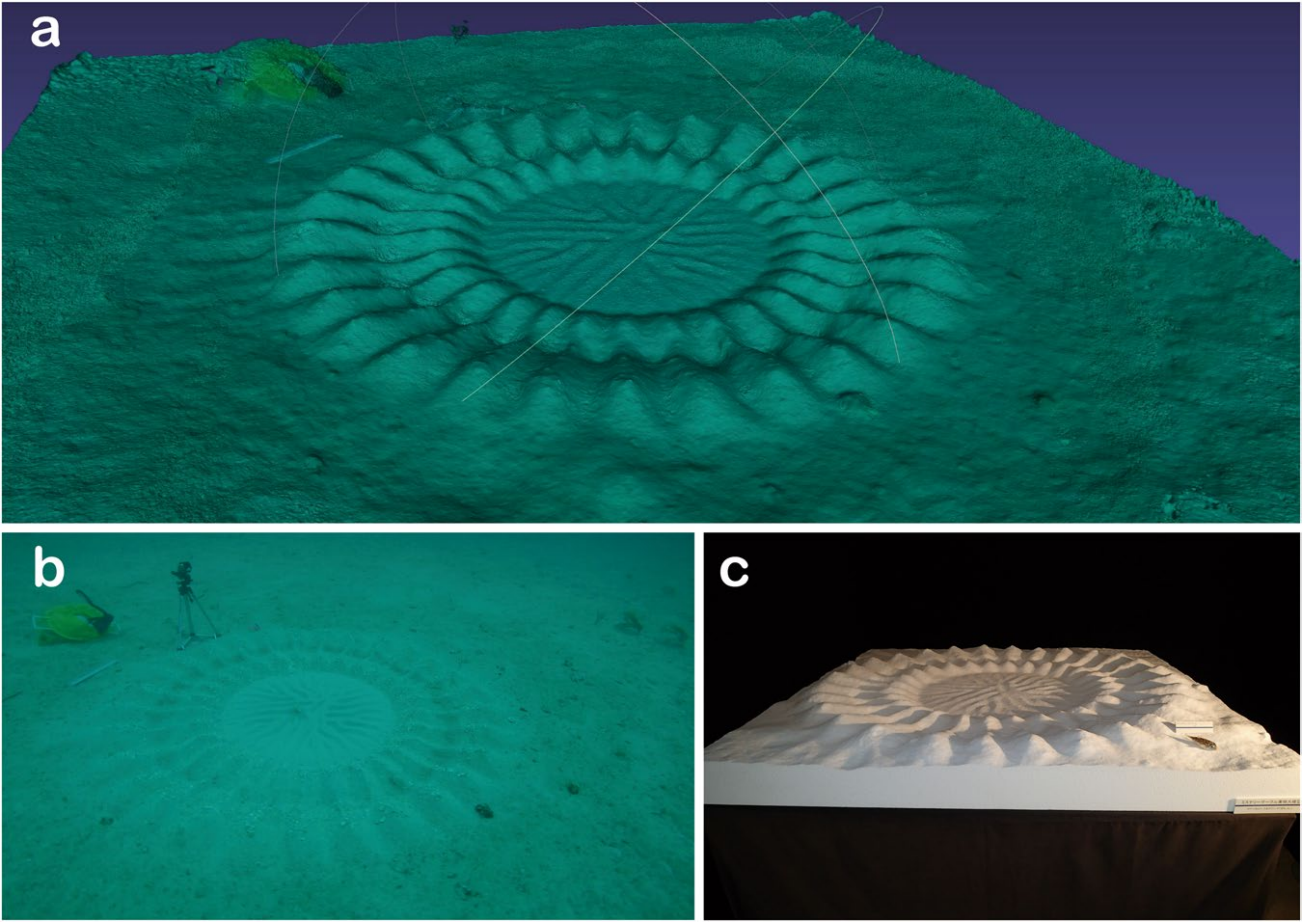
“Mystery circle” constructed by a white-spotted pufferfish (*Torquigener albomaculosus*). 3D model displayed on a computer (**a**), one of the video frames used to reconstruct the 3D model (**b**), and a Styrofoam model output in full size created using a 3D printer and the 3D data (**c**) for a specific mystery circle 20160615_K13.

Although data on the reproductive ecology and circle-construction behaviour of these pufferfish have been collected, many questions remain. Our interdisciplinary research currently has two themes: (i) theoretical studies on the logic of 3D-structure formation of the circular structure and (ii) ethological studies on the relationship between female mate choice and the features of the structure. To advance these studies, it is essential to collect quantitative data on the circular structure. Thus, we reconstructed 3D models of six completed mystery circles using a “structure from motion” (SfM) algorithm (Fig. 2).

On the other hand, the mystery circle constructed by the pufferfish may have potential applications in biomimetics similar to the structures constructed by termites and prairie dogs. To support the importance of its structural characteristics, it has been observed that the water passing through the valley upstream always gathers in the center of the structure, regardless of the direction of water flow^4^. Furthermore, particle size analysis of the sand forming the mystery circle has revealed that it has the function of extracting fine-grained sand particles from the valleys arranged radially to the outside and directing them to the center (Kawase, in prep.). The field of computational fluid dynamics, which makes full use of fluid dynamics technology, engineering knowledge, and computers, will logically clarify the characteristics of the 3D structure of the mystery circle we have reconstructed here. Shameem *et al*. reconstructed a 3D model of a mystery circle to explore the flow features with 2D computational fluid dynamic simulations^8^. Since our model has already been quantified as 3D data, computational fluid analysis can be immediately performed using this data, and the structural features of the mystery circle are expected to be applied in a wide range of fields, such as architecture and engineering, via biomimetics.

## Methods

During the breeding season, male *T. albomaculosus* individuals repeatedly construct a mystery circle, spawn with females, and care for the eggs at the circle’s central area in a semilunar cycle; the structures are completed on the same day in the same area^7^. Thus, six mystery circles (K1, K2, and K11–14) synchronously completed on June 15, 2016, at two locations on the seabed (depths of 13–16 m and 21–25 m, respectively) off Katetsu, Amami-Oshima Island, Japan (28° 07′ 53″ N, 129° 20′ 36″ E), were recorded in the following manner.

Each of the mystery circles was recorded using a video camera (GoPro Hero 4 Black Edition; GoPro, Inc., CA, USA) with a quality of 4 K and at 15 frames per second (fps). First, a 30-cm ruler was set on the outside of the mystery circle as an indicator of size. Next, the diver swam around the mystery circle for ~1 min from a distance of ~3 m from the center to record the entire circle and the ruler.

Having recorded the video, we reconstructed the 3D shape of each nest using the SfM algorithm. In total, ~ (900 ( = 15 fps × 60 s) video frames were acquired for each nest. The video frames were fed into the SfM software Agisoft, PhotoScan (Agisoft LLC, St. Petersburg, Russia), ver1.4.2, which was run on a PC (CPU: Intel Core i7-6900K 3.2 GHz; RAM: 128 GB; OS: Windows 7). We adjusted the parameters of the software to maximize the accuracy of the 3D shape of the reconstructed nest. If the software froze due to insufficient memory, we decimated the video frames (see Technical Validation).

## Data Records

The 3D models of the six mystery circles (original-size and reduced-size files) and original video clips (Table 1) are available from figshare^9^. Mean and range of the outer radius, inner radius, and height difference of six completed mystery circles measured 1020 (955–1077) mm, 375 (333–411) mm, 106 (97–113) mm, respectively.

**Table 1:** File name of the original movie, reconstructed 3D model, and reduced-size 3D model of six completed “mystery circles” constructed by white-spotted pufferfish (*Torquigener albomaculosus*) on the sandy seabed off Amami-Oshima Island, Japan, on June 15, 2016. All files are stored in figshare at 10.6084/m9.figshare.c.5783702.v1^9^.

Mystery circle ID	Reconstructed 3D model	Reduced-size 3D model	Original movie
20160615_K1	20160615_K1-3d_scaled.ply	20160615_K1-3d_scaled_reduced.ply	20160615_K1_3D.mp4
20160615_K2	20160615_K2-3d_scaled.ply	20160615_K2-3d_scaled_reduced.ply	20160615_K2_3D.mp4
20160615_K11	20160615_K11-3d_scaled.ply	20160615_K11-3d_scaled_reduced.ply	20160615_K11_3D.mp4
20160615_K12	20160615_K12-3d_scaled.ply	20160615_K12-3d_scaled_reduced.ply	20160615_K12_3D.mp4
20160615_K13	20160615_K13-3d_scaled.ply	20160615_K13-3d_scaled_reduced.ply	20160615_K13_3D.mp4
20160615_K14	20160615_K14-3d_scaled.ply	20160615_K14-3d_scaled_reduced.ply	20160615_K14_3D.mp4

## Technical Validation

In general, it is necessary to balance the trade-off between computational time and reconstruction accuracy when using the SfM algorithm. Accuracy can be maximized by increasing the number of input images captured from different viewpoints. However, in actual usage scenarios, the number of input images must be limited due to computational memory and time restrictions. In our analysis, we experimentally determined the number of input images (388–991) required for each nest. Specifically, we began with the maximum number of images and gradually reduced this number every time the software froze or the processing time was too long.

Shameem *et al*. reconstructed a 3D model of a mystery circle for computational fluid dynamic simulations^8^; however, the 3D data is not published anywhere. They used a commercially available video and obtained a sequence of 23 images for the 3D reconstruction, where estimated values of the global scale were used. In contrast, we shot original videos of mystery circles on the premise of reconstructing 3D models of mystery circles. Therefore, the absolute scale is known, and it was possible to extract sufficient and appropriate video frames from the movies shot from various directions at various angles. The six 3D models we reconstructed were the mystery circles which were completed in the same area on the same day by six males. Therefore, it was possible to compare the features in 3D models between individuals under the same conditions.

## Usage Notes

Each original 3D model has a file size of ~1 GB. Therefore, a high-end computer may be required to appropriately display the files with 3D software; otherwise, the operation may be very slow, in which case a smaller processed file could be used. Alternatively, a new 3D model could be constructed using the original video clips that were used to construct the 3D models described in this study.

## Data Availability

No code has been provided because the 3D data were reconstructed using commercial SfM software package of Agisoft PhotoScan (Agisoft LLC, St. Petersburg, Russia), ver1.4.2.
